# Correction: Automatic detection of expressed emotion from five-minute speech samples: Challenges and opportunities

**DOI:** 10.1371/journal.pone.0338220

**Published:** 2025-12-04

**Authors:** Bahman Mirheidari, André Bittar, Nicholas Cummins, Johnny Downs, Helen L. Fisher, Heidi Christensen

The Funding statement for this article is incorrect. The correct Funding statement is as follows: This project was funded by the Psychiatry Research Trust (https://www.psychiatryresearchtrust.co.uk/) [39C] and UK Medical Research Council (MRC, https://www.ukri.org/councils/mrc/) [MR/X002721/1] to JD. The E-Risk Study is funded by the UK MRC [G1002190 and MR/X010791/1] to HLF. NC is part funded by the National Institute for Health and Care Research (NIHR, https://www.nihr.ac.uk) Maudsley Biomedical Research Centre at South London and Maudsley NHS Foundation Trust and King’s College London. JD received support from a NIHR Clinician Scientist Fellowship [CS-2018-18-ST2–014] and Psychiatry Research Trust Peggy Pollak Research Fellowship in Developmental Psychiatry. HLF is part supported by the Economic and Social Research Council (ESRC, https://www.ukri.org/councils/esrc) Centre for Society and Mental Health at King’s College London [ES/S012567/1]. The views expressed are those of the authors and not necessarily those of the MRC, ESRC, NIHR, the Department of Health and Social Care, the University of Sheffield, or King’s College London. The funders had no role in study design, data collection and analysis, decision to publish, or preparation of the manuscript.
